# The impact of China’s employee basic medical insurance outpatient pooling scheme on outpatient healthcare utilization among middle-aged adults

**DOI:** 10.1371/journal.pone.0350183

**Published:** 2026-05-28

**Authors:** Xinjuan Zhou, Xinrui Li, Haiyi Chen, Jing Deng

**Affiliations:** Department of Public Administration, College of Public Health, Chongqing Medical University, Chongqing, China; Dayeh University, TAIWAN

## Abstract

**Background:**

This study evaluates the association between China’s Employees’ Basic Medical Insurance (EBMI) outpatient pooling policy and outpatient service utilization among middle-aged insured individuals (aged 45–60). By analyzing outpatient service use, visit frequency, and out-of-pocket (OOP) expenditures, the study documents utilization patterns associated with the policy and explores their implications for healthcare-seeking behavior among middle-aged enrollees, providing evidence that may inform future adjustments to outpatient coverage design.

**Methods:**

Using cross-sectional data from the 2018 wave of the China Health and Retirement Longitudinal Study (CHARLS), this study employed a logistic regression model to examine the effect of the outpatient pooling policy and different levels of outpatient benefit coverage on the probability of outpatient visits. A zero-inflated negative binomial regression model was used to analyze the impact on the frequency of outpatient visits, and a Tobit model was applied to assess its effect on out-of-pocket outpatient expenditures. Although the analysis relies on 2018 pilot data, the core institutional mechanisms of outpatient pooling were retained in the post-2021 national framework, suggesting that behavioral responses observed in pilot regions may still be informative under the current reform framework.

**Results:**

Implementation of the outpatient pooling policy was associated with a 5.0 percentage points higher probability of outpatient visits (*p* < 0.05) and a significantly higher visit frequency (IRR = 1.80, *p* < 0.01). Predictive estimates indicate that expected out-of-pocket expenditure in policy regions was approximately 48% higher than in non-policy regions (*p* < 0.05). Regions with higher levels of outpatient benefit coverage exhibited stronger utilization responses. In addition, individuals with multiple chronic conditions demonstrated significantly greater outpatient healthcare utilization.

**Conclusions:**

The EBMI outpatient pooling policy was associated with higher outpatient service utilization, suggesting improved access to previously unmet outpatient healthcare needs. However, additional adjustments may be required to better address the needs of patients with chronic conditions and to strengthen primary healthcare resources, thereby contributing to improvements in equity and efficiency.

## Introduction

China’s social health insurance system is undergoing a gradual but important shift from a model historically centered on inpatient care to one that integrates and elevates the role of outpatient services. Within the Employees’ Basic Medical Insurance (EBMI), this legacy manifested as robust pooled funding for hospitalizations, while general outpatient expenses were largely relegated to individual medical savings accounts or out-of-pocket (OOP) payments. This institutional design, with its pronounced hospitalization bias, has become increasingly misaligned with the evolving health needs of an aging population and the growing burden of chronic diseases, which demand frequent and continuous outpatient management. The limitations of personal accounts—often insufficient for actual needs yet paradoxically accumulating idle balances—have highlighted inefficiencies and equity concerns within the system.

To address these structural gaps, China initiated a nationwide reform of the outpatient pooling mechanism within EBMI in 2021. This reform established a separate pooled fund for general outpatient services, covering eligible expenses at designated institutions through defined parameters such as deductibles, reimbursement ratios, and annual caps [1,2]. It is important to clarify that the policy effects examined in this study primarily derive from local outpatient pooling schemes piloted across various regions prior to the 2021 national mandate. These local pilots, shaped by regional economic conditions, fund capacities, and fiscal resources, exhibited significant variation in coverage levels, generating variation in policy intensity across regions. This variation provides a valuable quasi-experimental setting for assessing the micro-level impact of the policy based on regional disparities.

A critical distinction must be made between the general outpatient pooling system studied here and China's long-standing Outpatient Chronic and Special Diseases (OCSD) coverage policy. The OCSD policy targets specific, severe chronic conditions (e.g., diabetes with complications) with strict diagnostic criteria and high coverage levels. In contrast, the general outpatient pooling system requires no such disease-specific certification. It casts a wider net, covering a broad insured population, including those who require long-term management for conditions (e.g., pre-diabetes, controlled hypertension) that may not yet meet the stringent OCSD criteria but still necessitate regular outpatient care. By filling this coverage gap for a substantial at-risk population, the general outpatient pooling system forms a foundational layer of China's outpatient security framework.

This study focuses on middle-aged enrollees for two key reasons. First, this life stage is critical for the onset and progression of chronic diseases, where outpatient services transition from sporadic use to a primary mode of continuous healthcare demand. Second, middle-aged individuals, often bearing significant familial and economic responsibilities, exhibit high sensitivity to out-of-pocket costs and policy incentives when making healthcare decisions [3–7]. This group thus represents both a core driver of outpatient demand and an ideal cohort for evaluating the behavioral impacts of the outpatient pooling reform.

The existing literature provides a foundation through three main streams. First, theoretical frameworks (e.g., Andersen’s model, price elasticity of demand, and moral hazard) suggest that reducing cost-sharing enhances healthcare utilization, particularly for price-elastic outpatient services [8–13]. Second, international comparative studies (e.g., on Medicare Part D in the U.S., and systems in Japan and Southeast Asia) show that expanding outpatient coverage improves access and disease management but may also increase costs and utilization risks, offering valuable lessons for China [14–19]. Third, empirical studies within China have begun to document the effects of outpatient coverage expansion, though research has predominantly focused on macro-level analyses or elderly populations [20–23]. Several important gaps remain. Specifically, there is a lack of dedicated analysis targeting the middle-aged cohort—a group central to both the labor force and chronic disease management. Furthermore, few studies systematically examine policy effects across both the extensive margin (the decision to seek care) and the intensive margin (the frequency and cost of care). Finally, analyses leveraging regional policy variation to investigate how different coverage levels modulate these effects are scarce. To address these gaps, this study employs nationally representative data to empirically assess the impact of the outpatient pooling policy on the utilization and out-of-pocket expenses of middle-aged enrollees. By exploiting the variation in the timing and design of local pilot policies, we examine how different policy intensities shape these outcomes. Our findings aim to provide micro-level evidence that may contribute to ongoing policy discussions.

## Materials and methods

### Materials

This study uses a cross-sectional design based on data from the 2018 wave of the China Health and Retirement Longitudinal Study (CHARLS), which covers 28 provinces (including municipalities and autonomous regions) across China. The CHARLS dataset mainly targets individuals aged 45 and above and provides rich information on demographic characteristics, health status and functioning, medical insurance and healthcare, and medical expenditures [24], thus offering strong data support for evaluating the impact of the EBMI outpatient pooling policy on healthcare utilization among enrollees. This study is a retrospective secondary analysis of publicly available data from the CHARLS. The CHARLS project received ethical approval from the Institutional Review Board at Peking University (IRB00001052–11015), and all participants provided informed consent during the original data collection. The researchers obtained the de-identified dataset from the CHARLS official website (http://charls.pku.edu.cn) on February 22, 2025. This study did not obtain or use any personally identifiable information and did not require additional ethical approval.

### Definition of the study population

This study focuses on enrollees of the EBMI aged 45–60. This age range was selected to capture the “middle-aged” population in China, a group defined by the critical life stage preceding the official old age threshold of 60 years [25].

The EBMI system in China provides continuous coverage to individuals from their working years into retirement. Therefore, our study population, drawn from EBMI enrollees aged 45–60, legitimately includes both active employees and those who have retired early. We have controlled for retirement status in all regression models to account for potential heterogeneity in healthcare utilization between these subgroups.

The sample selection and data processing procedures were as follows.

Based on the CHARLS 2018 questionnaire item EA001_W4 (“Are you currently enrolled in any of the following medical insurance schemes?”), individuals covered by the Urban Employees Basic Medical Insurance were identified. The age range was further restricted to those aged 45–60 years. Respondents were then categorized into “policy reform areas” and “non-reform areas” according to whether their city had implemented the EBMI outpatient pooling policy.

For the “retirement status” variable, if missing, information from other survey waves was used to supplement missing values. Specifically, if a respondent had ever been recorded as “retired” prior to 2018, they were treated as retired in 2018; if recorded as “not retired” in subsequent waves, they were assumed to be not retired in 2018. Observations with missing values in other key variables after this supplementation were excluded.

Continuous variables such as outpatient expenditures and total household income were winsorized at the 1st and 99th percentiles to mitigate the impact of extreme values. The variable for out-of-pocket outpatient expenditures was log-transformed to further control for skewness. Following the above selection and cleaning procedures, a total of 1,099 valid observations were retained for analysis.

### Methods

This study evaluates the impact of the EBMI outpatient pooling policy on outpatient service utilization among enrollees from three perspectives. First, it examines whether outpatient visits occur; second, it explores outpatient behavior as represented by the frequency of visits; and finally, it assesses the effect of the policy on individuals’ financial burden. Based on the statistical characteristics of each dependent variable, appropriate econometric methods were employed for empirical analysis.

The dependent variable “whether an outpatient visit occurred” (yes = 1, no = 0) is a binary variable for which a linear relationship with the explanatory variables is not assumed. Therefore, a logistic regression model was applied to analyze the effect of the outpatient pooling policy on the probability of having an outpatient visit among enrollees. The model is specified as follows:


$$\begin{array}{c} P\left(Y=\text{1}|X\right)=\frac{\text{1}}{\text{1}+e^{-\left(\beta_{\text{0}}+\beta_{\text{1}}X_{\text{1}}+\beta_{\text{2}}X_{\text{2}}+\ldots +\beta_{p}X_{p}\right)}} \end{array}$$ (1)

where *P*(*Y* = 1∣*X*) represents the probability that the dependent variable *Y* equals 1 (i.e., an outpatient visit occurs) given the explanatory variables *X*_*1*_, *X*_*2*_*, …, X*_*p*_ denote the explanatory variables, including whether the outpatient pooling policy was implemented, individual sociodemographic characteristics, health status, and other covariates.

The dependent variable “number of outpatient visits” is a non-negative count variable whose variance exceeds its mean, with a high proportion of zero values (85.9%), indicating overdispersion and excess zeros. Accordingly, this study uses a zero-inflated negative binomial regression model (ZINB) to analyze the impact of the policy on the number of outpatient visits. The model comprises two parts:


$$\begin{array}{c} \text{Pr}\left(Y_{i}=\text{0}\right)=\pi_{i}+\left(\text{1}-\pi_{i}\right)\cdot {\left(\frac{r}{\mu_{i}+r}\right)}^{r},if\;y_{i}=\text{0} \end{array}$$ (2)

$$\begin{array}{c} \text{Pr}\left(Y_{i}=y_{i}\right)=\left(\text{1}-\pi_{i}\right)\cdot \left(\frac{\Gamma \left(r+y_{i}\right)}{\Gamma (r)\;y_{i}!}\cdot {\left(\frac{\mu_{i}}{\mu_{i}+r}\right)}^{y_{i}}\cdot {\left(\frac{r}{\mu_{i}+r}\right)}^{r}\right),if\;y_{i}>\text{0} \end{array}$$ (3)

Equation (2) represents the zero-inflation part, and Equation (3) specifies the count process. Here, *y*_*i*_ is the observed number of outpatient visits for individual *i, μ*_*i*_ is the mean parameter of the negative binomial distribution, *r* denotes the dispersion parameter, and *π*_*i*_ indicates the probability of structural zeros.

The dependent variable “out-of-pocket outpatient expenditures” is a non-negative continuous variable with approximately 85.99% of its distribution at zero, exhibiting left censoring and significant skewness. To address this, out-of-pocket expenditures were log-transformed, and a Tobit regression model was used to analyze the effect of the outpatient pooling policy on out-of-pocket costs. To accommodate zero expenditures, we used log (OOP + 1) as the dependent variable in the Tobit model, with left-censoring at 0. The Tobit model is defined as:


$$\begin{array}{c} \overset{*}{\underset{i}{y}}=X_{i}\beta +\epsilon_{i},\epsilon_{i}\sim N\left(\text{0},\sigma^{\text{2}}\right) \end{array}$$ (4)

$$\begin{array}{c} y_{i}=\left\{ \begin{array}{c} \overset{*}{\underset{i}{y}},\overset{*}{\underset{i}{y}}>\text{0}\\ \text{0},\overset{*}{\underset{i}{y}}\leq \text{0} \end{array}\right.\; \end{array}$$ (5)

where *y*_i_^*^ is the latent variable representing potential outpatient costs, *y*_*i*_ is the actual observed value, and *X*_*i*_ includes the policy variable and relevant control variables.

### Variable selection

The core dependent variables in this study are indicators of outpatient healthcare utilization, which include both the choice of seeking outpatient services and the financial aspects of outpatient care utilization [26,27]. Based on the actual data available in the CHARLS database, these variables correspond to whether an outpatient visit occurred, the number of outpatient visits, and out-of-pocket outpatient expenditures. These indicators comprehensively capture different dimensions of outpatient healthcare utilization: “whether an outpatient visit occurred” reflects the occurrence of outpatient service use; “number of outpatient visits” represents the frequency of outpatient visits; and “out-of-pocket expenditures” measure the financial burden associated with outpatient services. The dependent variable “number of outpatient visits” is derived from the CHARLS questionnaire item [ED005], which specifically asks about visits for treatment of illness or injury, thereby excluding preventive health check-ups.

The key independent variables are whether the outpatient pooling policy was implemented and the level of outpatient benefit coverage. According to existing literature [20] and local policy documents for each sample region, a total of 30 regions had implemented the EBMI outpatient pooling policy by 2018. Based on the policy design features—specifically the annual deductible (“starting line”) and the maximum reimbursement limit (“ceiling line”)—the implementing regions were categorized into five groups: cities with an annual ceiling exceeding RMB 10,000 or with no ceiling are defined as “high benefit level”; cities without a deductible and with an annual ceiling over RMB 1,000 are classified as “relatively high benefit level”; cities without a deductible but with an annual ceiling below RMB 1,000 are classified as “moderate benefit level”; cities with a deductible and an annual ceiling over RMB 1,000 are defined as “relatively low benefit level”; and cities with both a deductible and an annual ceiling below RMB 1,000 are defined as “low benefit level.” The threshold values were not taken directly from a single policy document but were constructed to standardize heterogeneous regional policy parameters into comparable analytical categories. Specifically, official regional policy documents report a wide range of deductible and annual ceiling values that vary substantially across pilot cities. To capture meaningful institutional differences while preserving cross-region comparability, we grouped policy parameters into structured ranges reflecting distinct benefit regimes rather than using raw monetary values. These ranges were determined based on the empirical distribution of policy parameters observed across pilot regions and commonly reported policy design intervals in prior policy summaries. Therefore, the classification represents standardized institutional tiers rather than literal policy cut-off points. This approach is consistent with comparative policy analyses in which heterogeneous local parameters are harmonized into analytical categories to reflect substantive differences in incentive structures while avoiding spurious precision from nominal thresholds. Across pilot regions, annual reimbursement ceilings ranged from several hundred RMB to over 10,000 RMB, while deductibles varied from zero to several thousand RMB, supporting the use of categorical grouping to capture meaningful institutional differences. Accordingly, the categorization should be interpreted as institutional tiers capturing relative generosity, rather than exact monetary comparators across provinces. The classification of pilot cities by benefit level is presented in Table 1. As an additional robustness test, we also collapsed the five categories into three broader tiers, which yielded consistent results (Table 5).

**Table 1:** Levels of Outpatient Benefit Coverage.

Benefit Level	Pilot Cities
Low	Maoming, Taizhou (JS), Weifang, Binzhou
Relatively Low	Xuzhou, Lianyungang, Shijiazhuang, Hohhot, Jinan, Yancheng, Jiaxing, Suzhou, Tianjin
Moderate	Chaozhou, Jiangmen, Zhangzhou, Fuzhou, Dalian
Relatively High	Lishui, Shenzhen, Qingdao, Huzhou, Guangzhou
High	Beijing, Jingmen, Shanghai, Hangzhou, Ningbo, Qingyuan, Taizhou (ZJ)

To control for individual heterogeneity in outpatient utilization, the study includes a set of control variables covering demographic, household, and health characteristics [28–36]. The selection of independent variables in this study was guided by Andersen’s Behavioral Model of Health Service Use. According to this framework, healthcare utilization is determined by three categories of factors: (1) predisposing factors, such as age, gender, and education; (2) enabling factors, such as household income and place of residence, which influence individuals’ ability to obtain care; and (3) need factors, such as self-rated health and the number of chronic conditions, which reflect perceived and evaluated health needs. This model has been widely applied in studies using CHARLS and other large-scale health surveys, and it provides a theoretical basis for the variables included in our regression models. Demographic characteristics include age, gender, educational attainment, and retirement status (considering the characteristics of middle-aged women and those who retire early). Household characteristics include per capita monthly household income and place of residence. Per capita household income was used rather than individual annual income for two main reasons. First, in the CHARLS dataset, individual income information is often incomplete or inconsistently reported, especially among informal workers, unemployed individuals, and retirees who are still covered under EBMI. In contrast, household income is more reliably collected and has been widely used in previous CHARLS-based studies. Second, in China, outpatient medical expenses are commonly financed at the household level. Thus, per capita household income better reflects an individual’s actual economic capacity and is more appropriate for analyzing healthcare utilization. This approach aligns with established practices in health services research. Health characteristics include self-rated health status and the number of chronic conditions. The count of chronic conditions is a well-established measure of comorbidity burden and is a strong predictor of healthcare utilization in health services research [37,38]. Participants with chronic conditions were not excluded, as assessing the policy's impact across varying health statuses, particularly on those with multimorbidity, was a key objective of this study.

## Results

### Descriptive statistics

As shown in Table 2, approximately 14.1% of enrollees in the sample reported having an outpatient visit in the past month, with an average of 0.323 outpatient visits per patient. Regarding policy implementation, 31.67% of the respondents lived in areas where the EBMI outpatient pooling policy had been implemented, while the remaining 68.33% were in areas where the policy had not yet been adopted at the time of the survey. Among the regions with the outpatient pooling policy in place, the majority were classified as having relatively low levels of outpatient benefit coverage.

**Table 2:** Variable Classification and Descriptive Statistics.

Variable	Category	Full Sample (N = 1099)	Reform Areas (N = 348)	Non-Reform Areas (N = 751)
Outpatient Visit	Yes = 1	155 (14.10%)	59 (16.95%)	96 (12.78%)
	No = 0	944 (85.90%)	289 (83.05%)	655 (87.22%)
Number of Outpatient Visits	Monthly visits per patient	0.323	0.417	0.28
Log Out-of-Pocket Expenditure	Log of monthly out-of-pocket costs	0.819	0.968	0.75
Outpatient Pooling Policy	Yes = 1	348 (31.67%)	348 (100.00%)	—
	No = 0	751 (68.33%)	—	751 (100.00%)
Benefit Level	No pooling = 0	751 (68.33%)	—	751 (68.33%)
	Low = 1	32 (2.91%)	32 (9.20%)	—
	Relatively Low = 2	153 (13.92%)	153 (43.97%)	—
	Moderate = 3	25 (2.27%)	25 (7.18%)	—
	Relatively High = 4	47 (4.28%)	47 (13.51%)	—
	High = 5	91 (8.28%)	91 (26.15%)	—
Age	Patient age	53.162	53.066	53.206
Gender	Male = 1	586 (53.32%)	178 (51.15%)	408 (54.33%)
	Female = 0	513 (46.68%)	170 (48.85%)	343 (45.67%)
Education	Below Primary = 1	40 (3.64%)	22 (6.32%)	18 (2.40%)
	Primary = 2	345 (31.39%)	123 (35.34%)	222 (29.56%)
	Middle School = 3	238 (21.66%)	83 (23.85%)	155 (20.64%)
	High School & Above = 4	476 (43.31%)	120 (34.48%)	356 (47.40%)
Retirement Status	Yes = 1	389 (35.40%)	121 (34.77%)	268 (35.69%)
	No = 0	710 (64.60%)	227 (65.23%)	483 (64.31%)
Monthly Household Income	Per capita income (10,000 RMB)	0.348	0.388	0.329
Residence	Rural = 1	179 (16.29%)	71 (20.40%)	108 (14.38%)
	Urban = 0	920 (83.71%)	277 (79.60%)	643 (85.62%)
Self-Rated Health	Very Poor = 1	18 (1.64%)	2 (0.57%)	16 (2.13%)
	Poor = 2	83 (7.55%)	24 (6.90%)	59 (7.86%)
	Fair = 3	543 (49.41%)	180 (51.72%)	363 (48.34%)
	Good = 4	263 (23.93%)	69 (19.83%)	194 (25.83%)
	Very Good = 5	192 (17.47%)	73 (20.98%)	119 (15.85%)
Number of Chronic Diseases	0 = 0	317 (28.84%)	117 (33.62%)	200 (26.63%)
	1 = 1	278 (25.30%)	87 (25.00%)	191 (25.43%)
	2 = 2	224 (20.38%)	72 (20.69%)	152 (20.24%)
	≥ 3 = 3	280 (25.48%)	72 (20.69%)	208 (27.70%)

In terms of demographic characteristics, the average age of respondents was 53.16 years, with males accounting for 53.32% of the sample. Most respondents had an education level of high school or above, and 35.4% of the sample were already retired. For household characteristics, the mean per capita monthly household income was RMB 3,480, and the majority of respondents resided in urban areas. Regarding health status, most respondents rated their health as “fair,” and a substantial proportion reported having at least one chronic disease.

### Regression results

The regression results are shown in Table 3. They indicate that implementation of the outpatient pooling policy was associated with a significantly higher probability of outpatient visits among EBMI enrollees. Marginal effect estimates show that policy exposure increased the probability of having an outpatient visit by approximately 5.0 percentage points (*p* < 0.05).

**Table 3:** Baseline Regression Results: Logit, ZINB, and Tobit Models.

Variable	Outpatient Visit (Logit)		Outpatient Visits (Frequency) (ZINB)		Out-of-Pocket Expenditures (Tobit)	
Implementation of Outpatient Pooling Policy	0.452**	-	0.588***	-	2.007**	-
	(0.194)	-	(0.224)	-	(0.884)	-
Benefit Level (ref: Non-implementation)						
Low	-	-0.655	-	-1.223*	-	-0.238
	-	(0.753)	-	(0.665)	-	(0.325)
Relatively Low	-	0.533**	-	0.661**	-	0.296
	-	(0.252)	-	(0.301)	-	(0.188)
Moderate	-	-0.936	-	-1.822***	-	-0.252
	-	(0.897)	-	(0.662)	-	(0.205)
Relatively High	-	0.501	-	1.436**	-	0.337
	-	(0.428)	-	(0.646)	-	(0.348)
High	-	0.722**	-	0.575*	-	0.530*
	-	(0.298)	-	(0.301)	-	(0.271)
Age	0.004	0.003	0.028	0.040	0.020	0.002
	(0.025)	(0.025)	(0.035)	(0.035)	(0.119)	(0.018)
Gender (Male=1)	-0.512**	-0.513**	-0.954***	-0.971***	-2.615***	-0.381**
	(0.212)	(0.214)	(0.225)	(0.230)	(0.972)	(0.152)
Education (ref: Below Primary)						
Primary	0.876	0.270	2.450***	0.975*	4.140	1.187
	(0.728)	(0.461)	(0.729)	(0.546)	(3.281)	(2.136)
Middle School	0.311	-0.211	1.175	-0.248	1.324	-1.346
	(0.764)	(0.464)	(0.750)	(0.519)	(3.462)	(2.101)
High School & Above	0.763	0.205	1.524*	0.062	3.582	0.649
	(0.804)	(0.453)	(0.809)	(0.549)	(3.621)	(2.099)
Retirement Status (Retired=1)	-0.128	-0.146	-0.209	-0.317	-0.777	-0.154
	(0.240)	(0.242)	(0.304)	(0.269)	(1.116)	(0.173)
Household Income	0.205	0.013	0.330	0.303	0.101	0.011
	(0.325)	(0.027)	(0.372)	(0.390)	(0.127)	(0.019)
Place of Residence (Urban=0)	0.160	0.107	0.781**	0.683**	0.667	0.010
	(0.260)	(0.271)	(0.359)	(0.295)	(1.197)	(0.173)
Self-rated Health (ref: Very Poor)						
Poor	-0.719	-0.304	0.111	-0.770	-8.480***	-1.853
	(0.756)	(0.577)	(0.558)	(0.551)	(2.515)	(2.688)
Fair	-0.380	0.056	0.747	-0.0258	-6.701***	0.113
	(0.582)	(0.405)	(0.553)	(0.438)	(2.086)	(1.855)
Good	-0.954	-0.583	0.945	0.277	-10.10***	-3.110
	(0.721)	(0.681)	(0.781)	(0.655)	(3.179)	(2.801)
Very Good	0.000	0.387	2.274**	1.480**	-5.065	1.895
	0.000	(0.615)	(0.930)	(0.716)	(3.507)	(2.726)
Number of Chronic Conditions (ref: 0)						
1	0.315	-0.233	0.557	0.314	1.811	-1.215
	(0.508)	(0.502)	(0.673)	(0.598)	(2.281)	(2.289)
2	0.484	-0.018	0.148	0.0177	2.181	-0.425
	(0.516)	(0.458)	(0.619)	(0.553)	(2.286)	(2.075)
≥ 3	1.179**	0.631	0.516	0.374	5.502**	2.666
	(0.548)	(0.497)	(0.679)	(0.567)	(2.381)	(2.257)
Constant	0.077	0.115	-1.107	-1.554	0.120	2.300**
	(1.319)	(1.325)	(1.593)	(1.625)	(6.234)	(0.953)
Observations	1,099	1,099	1,099	1,099	1,099	1,099

Standard errors in parentheses.

* *p* < 0.05.

** *p* < 0.01.

*** *p* < 0.001.

Regarding benefit design, both relatively low and high levels of outpatient coverage were significantly associated with a higher likelihood of outpatient utilization (*p* < 0.05). Among control variables, males had a significantly lower probability of outpatient visits than females, and individuals with three or more chronic conditions were significantly more likely to seek outpatient care.

In terms of visit frequency, the zero-inflated negative binomial model indicates that the outpatient pooling policy was associated with a significantly higher visit rate (IRR ≈ 1.80, *p* < 0.01), suggesting that enrollees in policy regions had approximately 80% more outpatient visits than those in non-policy regions. In addition, males had significantly lower visit rates than females (*p* < 0.01); individuals with primary education had higher visit frequency; urban residents showed significantly higher utilization than rural residents (*p* < 0.05); and respondents reporting very good health also had higher visit rates (*p* < 0.05).

With respect to financial burden, predictive estimates from the Tobit model indicate that expected out-of-pocket expenditure was approximately 48% higher among individuals living in policy regions than among those in non-policy regions (*p* < 0.05). Higher benefit levels were also associated with higher expected out-of-pocket spending. In addition, males had significantly lower out-of-pocket costs than females (*p* < 0.01), self-rated health was negatively associated with expenditure, and individuals with three or more chronic conditions incurred significantly higher costs (*p* < 0.05).

### Robustness check

To assess the robustness of the baseline results, propensity score matching (PSM) was conducted using nearest-neighbor matching, and the average treatment effect on the treated (ATET) was estimated based on a matched sample of 1,099 observations (Table 4). The results indicate that the outpatient pooling policy remains significantly associated with a higher probability of outpatient visits and higher expected out-of-pocket expenditures, while the estimated effect on visit frequency remains positive but statistically insignificant. Compared with the baseline estimates, the effect sizes decrease slightly, suggesting that the main results may partly reflect observable selection differences. As matching improves covariate balance between treatment groups, these findings support the robustness of the baseline conclusions.

**Table 4:** Robustness Check: Propensity Score Matching (Nearest Neighbor, ATET).

Variable	Outpatient visit	Visit frequency	Log OOP expenditure
Policy implementation	0.058**	0.270	0.392**
	(0.029)	(0.229)	(0.166)
Controls	Yes	Yes	Yes
Observations	1,099	1,099	1,099

Standard errors in parentheses.

* *p* < 0.05.

** *p* < 0.01.

*** *p* < 0.001.

In addition to the robustness checks described above, we assessed the sensitivity of our results to the operationalization of benefit levels. Specifically, we collapsed the original five-category classification into three broader tiers (high, medium, and low) to reduce dependence on specific threshold definitions. Re-estimation of the models using this alternative grouping produced qualitatively similar patterns to the main specification. The direction and relative magnitude of estimated effects remained stable, indicating that our findings are not driven by the particular categorization of benefit levels. Detailed estimates are reported in Table 5. Results remain qualitatively unchanged when alternative grouping thresholds are applied.

**Table 5:** Robustness Check: Alternative Benefit Classification.

Benefit Tier	AME vs Low	Robust SE
High	0.026	0.043
Medium	−0.112*	0.047
Low (ref.)	—	—

Notes: AME = average marginal effect. Models include the same control variables as in the main specification. *p < 0.05.

## Discussion

### The outpatient pooling policy significantly releases and formalizes latent demand among middle-aged enrollees

Our empirical results consistently indicate that the outpatient pooling policy was associated with higher outpatient service utilization among insured individuals but did not reduce their out-of-pocket payments, resulting in a “dual increase in utilization and costs,” a pattern consistent with prior evidence [1,39]. This pattern may be partly explained by the policy’s effect of lowering the economic threshold for seeking care, thereby activating and formalizing previously suppressed outpatient demand. The consequent rise in the number of visits may contribute to an increase in total OOP expenditures [40,41].

By incorporating general outpatient expenses into the pooled fund, the reform changed enrollees’ cost expectations for outpatient care [42]. Compared to the previous reliance on personal medical savings accounts or full OOP payments, the policy alleviated immediate financial concerns, transforming outpatient visits from a decision often fraught with cost anxiety into a routine, institutionally-backed health management behavior with more predictable financial risk [22]. This shift occurs not by reducing the OOP amount per visit, but by lowering the decision threshold for whether to seek care, thereby increasing the total number of visits and cumulatively raising annual OOP expenditures. Under this mechanism, some previously unmet outpatient needs—such as early care for minor symptoms, regular chronic disease follow-ups, and medication adjustments—are activated and converted into actual visits [43]. These represent not new morbidity but pre-existing health management needs that were financially constrained. Their release is directly observable as a marked increase in outpatient visit volume and frequency [2].

Critically, this does not necessarily imply a significant increase in per-visit OOP costs. Under stable reimbursement rules, individuals continue to bear a relatively fixed co-payment ratio per visit. However, the cumulative effect of more frequent visits leads to higher annual OOP totals. Thus, the rise in OOP spending primarily reflects a “scale effect”—growth in total expenditure driven by expanded service volume, with the unit cost burden remaining relatively constant.

The increase in OOP payments should not be interpreted as a failure of the policy’s cost-protection goal, given the observational nature of this study. Moreover, the observed rise in utilization may partly reflect provider-level shifts or changes in service intensity, rather than improved access alone. Rather, it is a concomitant feature of improving outpatient accessibility and formalizing health management. These findings suggest that policy efforts may need to focus less on suppressing increased demand and more on improving the health returns from additional visits. This necessitates coordinated reforms in areas experiencing the strongest demand growth—particularly primary care and chronic disease management. Strengthening primary care capacity and advancing value-based payment models like capitation [44,45] can help steer the system away from volume-driven expansion and toward improved health outcomes, thereby achieving a more sustainable balance between cost growth and health gains.

### Heterogeneity in policy effects: The critical role of benefit design parameters

Beyond the aggregate “dual increase” pattern, this study identifies significant regional variation in the policy’s effect, consistent with earlier findings [40,41]. This heterogeneity is closely related to regional benefit design parameters—specifically the deductible and annual payment cap—set at the regional level [2,22]. This underscores that the specific design of coverage parameters is a critical institutional lever shaping the policy’s effectiveness in influencing behavior and achieving its intended outcomes.

These parameters systematically structure the incentive environment by defining distinct cost-sharing pathways [46]. The deductible sets the initial financial threshold for accessing coverage, while the payment cap defines the upper bound of an individual’s annual financial risk [13]. In regions with low deductibles and high caps, the system creates strong, consistent incentives: low upfront costs reduce barriers to initial and preventive care, while a high cap mitigates fears of catastrophic cumulative expenses. In this environment, enrollees are more likely to integrate outpatient care into sustained health management, allowing the policy’s full effects to materialize.

Conversely, regions with high deductibles or low caps generate weaker and more ambiguous incentives [41]. A high deductible leaves a substantial portion of initial costs fully OOP, negating the policy’s benefit for minor or episodic care. A low cap curtails the system’s risk-pooling function for those requiring frequent or long-term care, reinstating financial uncertainty. Under such designs, enrollees may perceive limited value from the coverage, leading to weaker behavioral responses.

Future reforms could consider more systematic parameter calibration rather than uniform benefit expansion. Specifically, deductible thresholds should be determined relative to predicted routine outpatient expenditure estimated from utilization data for typical enrollees so that initial access costs do not exceed anticipated preventive-care spending. Similarly, annual reimbursement ceilings should be set to cover the predicted yearly outpatient utilization of high-need populations, particularly individuals with multiple chronic conditions. Such rule-based actuarial principles allow parameter values to be locally calibrated while preserving a coherent national framework. This approach may help align benefit design with access, protection, and sustainability objectives.

### Multimorbidity and financial burden implications

Apart from regional variation, this study finds that middle-aged patients with multiple chronic conditions (multimorbidity) experience significantly higher outpatient utilization intensity and OOP burdens than those without chronic conditions, aligning with established literature [47–49]. This indicates that the policy’s burden-reducing effect is uneven across populations with differing health needs and is particularly constrained for those managing chronic diseases.

Unlike acute or episodic care, the healthcare needs of this group are defined by long-term, continuous, and stable nature [47]. Their care requires regular follow-ups, monitoring, and medication adjustments oriented around disease control. Consequently, their outpatient use is not sporadic but persistently high-frequency. In this context, even with improved reimbursement per visit, the cumulative effect of numerous annual visits substantially elevates total OOP expenses.

Under the current system, service delivery and payment remain organized around discrete, visit-based units focused on single diseases. Consequently, the continuous, integrated health management required by patients with multimorbidity is fragmented into multiple independent clinical encounters. Each visit triggers a separate billing event with its own cost-sharing obligations [50,51]. This “visit-centric” design may lead patients to accumulate repeated, cumulative OOP costs over the course of a year. Thus, their “high utilization–high OOP” profile stems not from overuse but from their legitimate, continuous care needs being disassembled and amplified by a fragmented system into a series of financially burdensome transactions.

Taken together, the evidence indicates that merely raising reimbursement rates or broadening covered services within the existing visit-based framework is insufficient to alleviate the economic burden on this population. Addressing this issue may require adjustments in how outpatient services are financed, particularly for patients with multimorbidity. Practically, this could involve piloting integrated care models for high-need patient groups (e.g., common multimorbidity clusters), anchored by family physician teams or medical alliances responsible for providing continuous, coordinated management. Payment should shift toward bundled or capitated models that cover the expected spectrum of care needs over a defined period, thereby dismantling the financial accumulation inherent in fee-for-service, visit-based billing. By mitigating the cost-amplifying effect of system fragmentation, the outpatient pooling policy can more effectively fulfill its risk-pooling and financial protection functions for populations with complex, chronic health needs. In practice, this implies that cost-sharing parameters for individuals with multimorbidity should be calibrated to their expected annual service needs, for example, through reduced or waived deductibles and higher reimbursement ceilings relative to predicted utilization levels. To operationalize such reforms, parameter design should follow actuarial principles linked to expected utilization intensity. For example, deductible levels for patients with multimorbidity could be calibrated so that annual cost-sharing does not exceed a fixed proportion of predicted routine outpatient expenditures. Payment models may further incorporate risk-adjusted capitation rates that scale with the number of chronic conditions, thereby aligning provider incentives with patients’ longitudinal care needs rather than visit volume. Establishing such rule-based parameter standards may allow policymakers to move from general coverage expansion toward more targeted benefit design for populations with persistently high healthcare needs.

### Policy continuity and external validity

A key concern in evaluating early-stage policy reforms is the temporal relevance of empirical findings. The data used in this study originates from 2018 and therefore precedes the nationwide rollout of China’s outpatient pooling reform in 2021. However, the national reform does not represent a discontinuous policy break, but rather an institutional consolidation and expansion of earlier pilot programs. The State Council’s 2021 guidance [52] explicitly emphasizes maintaining policy continuity and ensuring a smooth transition of benefits. Core elements of outpatient pooling—including pooled outpatient financing, restructuring of personal accounts, tiered cost-sharing arrangements, and strengthened primary care incentives—were already embedded in pre-2021 pilot schemes and subsequently formalized within the national framework.

Provincial and municipal implementation guidelines further illustrate this institutional continuity. For example, Shenzhen’s 2022 implementation regulations operationalize outpatient pooling through tiered reimbursement structures, annual expenditure caps, and primary care–oriented payment mechanisms such as capitation [53]. While specific numerical parameters vary across jurisdictions, the underlying architecture of the reform remains structurally comparable despite substantial local variation in implementation parameters. China’s outpatient insurance reform follows a decentralized and incremental trajectory in which regional experimentation informs national scaling, and local heterogeneity is an inherent feature of governance rather than a temporary anomaly.

From a policy evaluation perspective, early-stage pilot studies can identify behavioral mechanisms that persist through institutional continuity even as implementation parameters evolve. The objective of this study is not to estimate the ultimate long-run equilibrium effects of the reform, which would require extended longitudinal data, but to examine how outpatient pooling mechanisms shape healthcare utilization behavior. The pilot initiatives prior to 2018 established the fundamental mechanisms that the current decentralized framework has since consolidated and expanded. The utilization patterns documented in our empirical analysis therefore reflect mechanisms embedded in the current reform framework, supporting the continued policy relevance of our findings for interpreting ongoing outpatient insurance expansion. This institutional continuity is summarized in Table 6.

**Table 6:** Institutional Continuity of the Outpatient Pooling Reform.

Policy Dimension	Pre-2021 Pilot Phase (regional pilot implementation)	Post-2021 Reform (national framework + local parameters)	What Remains Comparable Across Periods
Institutional Architecture	Outpatient pooling arrangements explored in selected regions	Nationally mandated outpatient pooling architecture; provinces/municipalities set specific parameters	Shift toward pooled financing for outpatient services
Coverage Scope	Often partial or phased-in coverage for general outpatient services	Framework extends coverage to EBMI enrollees; scope/benefit generosity varies by locality	Expansion of outpatient coverage within EBMI
Cost-sharing Design	Deductibles, reimbursement rates, and caps set locally	Same instruments retained; values calibrated locally within national/provincial guidance	Use of deductibles/reimbursement/caps as core design instruments
Personal Account Reform	Mixed designs; varying shares and rules	Framework reshapes personal accounts with local implementation details	Rebalancing between personal accounts and pooled funds
Primary Care Incentives	Heterogeneous; some pilots introduced referral or gatekeeping incentives	Framework encourages primary-care orientation; implementation varies (e.g., designated providers, differential reimbursement)	Policy intent to steer care toward primary care
Provider Payment Reform	Fragmented pilots; mixed payment experiments	National guidance encourages mixed payment reforms; local rollout varies	Payment reform as an accompanying mechanism

Note: The national reform establishes a shared institutional framework while allowing substantial local variation in implementation parameters.

To further clarify comparability between the pre-2021 pilot phase and the post-2021 national framework, it is important to distinguish institutional mechanisms from parameter magnitudes. Across both periods, three core structural components remain consistent: (1) outpatient services are financed through pooled funds rather than individual medical savings accounts; (2) cost-sharing is governed by deductibles, reimbursement ratios, and annual ceilings; and (3) policy design incorporates incentives intended to guide patients toward primary care. These institutional features define the behavioral incentive structure faced by enrollees and constitute the primary mechanisms evaluated in this study. By contrast, differences between periods are concentrated in numerical parameter values (e.g., reimbursement rates or cap levels), which primarily affect the intensity rather than the direction of behavioral responses.

Because this study focuses on mechanism identification rather than long-run equilibrium effects, evidence from the pilot stage remains informative for understanding how outpatient pooling alters healthcare utilization decisions under the current policy framework. Because the core institutional incentives remain structurally consistent before and after the 2021 rollout, behavioral responses observed in pilot settings may still be informative, even though parameter magnitudes vary across regions and over time. Official policy documents indicate that the ranges of deductibles, reimbursement ratios, and annual caps observed in pilot programs fall within the parameter intervals adopted after the national reform. Although macroeconomic conditions have evolved since 2018, such changes are more likely to affect the overall level of healthcare demand rather than the underlying behavioral responses to insurance incentives.

## Limitations

One limitation concerns provider-level behavioral mechanisms. Although CHARLS records the type of outpatient institution visited, incorporating this variable into the harmonized analytic dataset would substantially reduce the effective sample size due to inconsistencies across variables after data cleaning. Consequently, the observed increase in OOP expenditure should be interpreted cautiously and cannot be attributed solely to improved access; alternative mechanisms such as provider-induced demand or shifts toward higher-level facilities remain plausible explanations. Future research using administrative claims data with complete provider identifiers could examine this mechanism more definitively. More broadly, causal inference remains limited because the analysis relies on observational cross-sectional data despite multiple robustness checks. Longitudinal datasets and stronger quasi-experimental designs, as well as analyses using more recent post-reform data, would help assess longer-term policy dynamics and strengthen causal identification. In addition, because the analysis relies on 2018 cross-sectional data, caution is warranted when extrapolating the magnitude of estimated effects to the current nationwide policy environment.
